# Histone modifications and depression: epigenetic mechanisms, therapeutic targets, and translational outlook

**DOI:** 10.3389/fgene.2025.1722453

**Published:** 2026-01-12

**Authors:** Liang-Zhen Lv, Zhao-Di Wang, Jia-jie Ren, Lu-Hao Li, Xian-bao Liu, Jia-li Li, Hui Zhu, Bei Jiang, Ya-peng Han, Xue-ming Zhou, Li Ren, Zhuo Chang

**Affiliations:** 1 Heilongjiang University of Chinese Medicine, Harbin, Heilongjiang, China; 2 Zhejiang Chinese Medical University, Hangzhou, China; 3 Yichun Central Hospital, Yichun, Heilongjiang, China; 4 First Affiliated Hospital of Heilongjiang University of Chinese Medicine, Harbin, Heilongjiang, China; 5 Shunde Women and Children’s Hospital, Guangdong Medical University, Foshan, Guangdong, China

**Keywords:** epigenetics, HDAC inhibitors, histone modifications, HMT inhibitors, major depressive disorder, neuroplasticity

## Abstract

Major depressive disorder (MDD) is a highly prevalent and heterogeneous psychiatric disorder shaped by the interplay of genetic, environmental, and epigenetic factors. Histone modifications, particularly acetylation and methylation, have emerged as critical regulators of chromatin dynamics and gene expression in stress adaptation, neuroplasticity, and emotional regulation. This review synthesizes current evidence linking dysregulated histone deacetylases (HDACs) and histone methyltransferases (HMTs) to impaired neuroplasticity, neuroinflammation, mitochondrial dysfunction, and hypothalamic–pituitary–adrenal (HPA) axis hyperactivity in depression. We further evaluate the therapeutic potential of HDAC and HMT inhibitors, highlight their effects beyond transcriptional control, and discuss peripheral epigenetic biomarkers as candidate tools for patient stratification and treatment prediction. Emerging technologies, including single-cell and spatial epigenomics as well as CRISPR-based epigenetic editing, are outlined as future avenues toward precision medicine. While isoform specificity, off-target effects, and translational heterogeneity remain major challenges, targeting histone modifications represents a promising strategy for next-generation antidepressant development.

## 1 Introduction

Major depressive disorder (MDD) is among the most prevalent psychiatric conditions, affecting over 280 million individuals globally (World Health Organization, 2025). Its global prevalence has risen by nearly 50% in the past three decades, making it a leading cause of disability and a major contributor to years lived with disability (Liu et al., 2020). Clinically, MDD is marked by persistent low mood, anhedonia, cognitive impairment, and physical symptoms such as sleep and appetite disturbances (Beurel et al., 2020). It is strongly associated with suicide and increases the risk of comorbid conditions including cardiovascular disease, diabetes, and inflammatory disorders (Mangione et al., 2022). These wide-ranging impacts underscore the urgent need for more effective and better-targeted therapeutic strategies.

Traditional genetic studies have identified heritable components of major depressive disorder (MDD), but they only partially account for its heterogeneity and sensitivity to environmental influences (Alshaya, 2022). Recent epigenetic research offers a broader framework by highlighting how gene–environment interactions dynamically shape disease vulnerability and treatment response. Epigenetics refers to heritable but reversible modifications in gene expression that occur without changes to the DNA sequence, primarily through mechanisms such as DNA methylation, histone modification, and non-coding RNA regulation (Aljabali et al., 2024; Mohammadi et al., 2023). These pathways serve as molecular bridges between environmental exposures—such as early-life stress or chronic adversity—and long-term changes in brain function (Czarny et al., 2021). In depression, altered epigenetic patterns have been observed in key genes related to neurodevelopment and neurotransmission, including the serotonin transporter gene (SLC6A4) and brain-derived neurotrophic factor (BDNF) (Jiang et al., 2019; Levy et al., 2018). Dysregulated microRNA expression is also linked to synaptic plasticity, neuroinflammation, and stress response (Ortega et al., 2021; Elhag and Al Khodor, 2023). Among these mechanisms, histone modifications—particularly acetylation and methylation—play a central role in mood-related gene expression and are emerging as viable therapeutic targets.

Histone modifications play a central role in the epigenetic regulation of depression by influencing chromatin structure and transcriptional control (Czarny et al., 2021; Nestler et al., 2016). In stress-related depression models, reduced histone acetylation in the prefrontal cortex and hippocampus correlates with downregulated expression of BDNF and CREB, key mediators of neuroplasticity and mood stabilization (Li et al., 2025). Changes in histone marks have been linked to neuroinflammation and neuronal injury, contributing to structural and functional alterations observed in depression (Yadav et al., 2024; Yuan et al., 2023; Zhu et al., 2021).

Chronic stress is associated with elevated HDAC activity in the hippocampus and prefrontal cortex, resulting in reduced expression of mood-related genes such as BDNF and CREB. Pharmacological inhibition of HDACs can reverse these molecular effects and produces antidepressant-like behavioral outcomes in animal models (Dai et al., 2023). The HDAC family consists of 18 isoforms with distinct biological functions, among which Class I and Class II isoforms appear to be most relevant to depression. However, the precise roles of individual HDAC isoforms remain to be fully elucidated. Collectively, these findings support the therapeutic potential of HDAC inhibition, which will be discussed in greater detail in later sections.

In summary, histone modifications—particularly those governed by HDAC activity—play a critical role in the epigenetic regulation of mood- and stress-related gene expression. Their reversible nature makes them compelling targets for therapeutic intervention in depression. The following sections will first introduce the basic biology of histone modifications, then examine their dysregulation in depressive states, and finally explore the therapeutic relevance of HDACs, HMTs, and their pharmacological inhibitors. Recent advances, persisting challenges, and future research directions in this rapidly evolving field will also be discussed.

**FIGURE 1:**
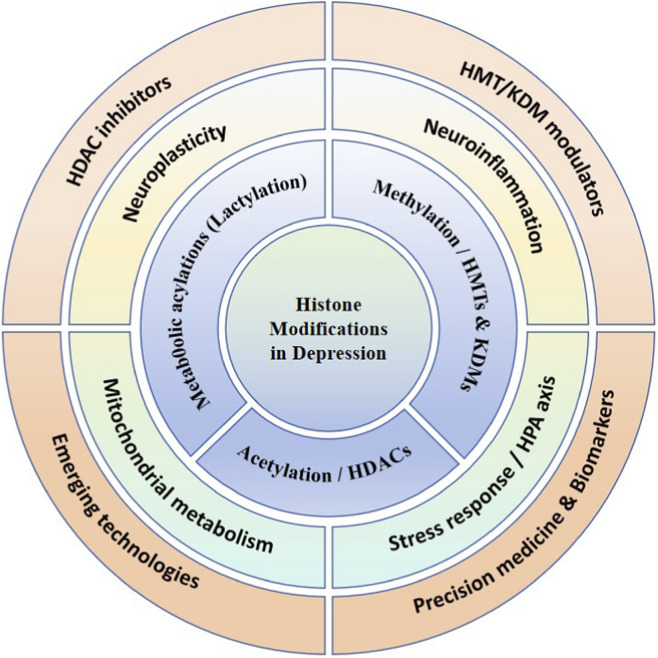
This figure summarizes a multilayered framework linking histone modifications to the pathophysiology and treatment of depression. The central core highlights histone modifications as a key epigenetic hub. The inner ring depicts major regulatory mechanisms, including histone acetylation (HDACs), histone methylation (HMTs/KDMs), chromatin accessibility, and metabolism-linked modifications such as histone lactylation. The middle ring illustrates downstream biological processes influenced by these mechanisms, including neuroplasticity, neuroinflammation, mitochondrial metabolism, and stress response via the HPA axis. The outer ring outlines translational perspectives, encompassing epigenetic modulators, precision medicine and biomarkers, and emerging technologies. Together, this framework integrates molecular regulation, functional consequences, and translational outlooks in depression.

The present review provides an integrated overview of histone modifications in the pathophysiology of major depressive disorder (MDD), with particular emphasis on the regulatory functions of histone deacetylases (HDACs) and histone methyltransferases (HMTs). To assist readers in navigating the conceptual framework, Figure 1 summarizes the multilayered organization of epigenetic regulation—from molecular modifiers to downstream biological processes and translational implications.

## 2 Histone modifications: biological mechanisms and functions

### 2.1 Nucleosome structure and histone composition

The nucleosome, the fundamental unit of chromatin, consists of ∼147 base pairs of DNA wrapped around a histone octamer composed of H2A, H2B, H3, and H4 (Arrieta and Vondriska, 2022). These highly conserved proteins compact genomic DNA while maintaining access for transcriptional regulation. The flexible N-terminal tails of histones protrude from the nucleosome and serve as major sites for post-translational modifications (PTMs), including acetylation, methylation, and phosphorylation (Millán-Zambrano et al., 2022; Le et al., 2023). These PTMs modulate nucleosome stability and chromatin accessibility, thereby influencing gene expression in a context-dependent manner. Such dynamic regulation is particularly relevant in the brain, where rapid transcriptional responses support neural plasticity and stress adaptation.

### 2.2 Histone acetylation

Histone proteins undergo multiple post-translational modifications (PTMs) that regulate chromatin structure and gene expression (Huang et al., 2014). These PTMs predominantly occur on the N-terminal histone tails and include acetylation, methylation, phosphorylation, and ubiquitination, forming combinatorial patterns often referred to as the “histone code,” which influences transcription in a context-dependent manner (Millán-Zambrano et al., 2022; Miziak et al., 2024; Jenuwein and Allis, 2001; Lam et al., 2019).

Among these modifications, histone acetylation is one of the most transcriptionally permissive marks. Acetylation is catalyzed by histone acetyltransferases (HATs), which neutralize lysine residues, loosen DNA–histone interactions, and promote chromatin accessibility. Major HAT families include GNAT, p300/CBP, MYST, and Rtt109 (Poziello et al., 2021; Sabnis, 2021; Di Cerbo and Schneider, 2013).

Although methylation also contributes to transcriptional regulation (e.g., H3K4me3 as an activating mark and H3K27me3 as a repressive mark) (Verma et al., 2022), acetylation provides a rapid and reversible mechanism particularly relevant for neural activity and stress-related gene expression. Together, these PTMs constitute a flexible regulatory system that can sustain or reshape transcriptional programs in response to environmental stressors, contributing to persistent epigenetic alterations observed in depression.

### 2.3 Enzymatic regulation of histone modifications

Histone modifications are dynamically regulated by coordinated enzyme systems commonly referred to as “writers,” “erasers,” and “readers,” which respectively deposit, remove, or recognize specific epigenetic marks (Kelly et al., 2018; Brancolini et al., 2022). This regulatory framework enables precise and reversible control of chromatin structure and transcriptional activity in response to developmental cues and environmental stimuli.

Histone deacetylases (HDACs), which function as key “erasers” of acetyl marks, are classified into five groups—Class I, Class IIa, Class IIb, Class III (sirtuins), and Class IV—based on sequence homology, subcellular localization, and cofactor dependence (Bahl et al., 2021; Bahl and Seto, 2021). Class I HDACs are predominantly nuclear and closely linked to transcriptional repression, whereas Class II HDACs shuttle between the nucleus and cytoplasm and are involved in activity-dependent gene regulation. Class III HDACs require NAD^+^ as a cofactor and connect chromatin regulation with cellular metabolic state, while HDAC11 (Class IV) shares features of both Class I and II enzymes.

In addition to HDACs, histone modifications are shaped by histone acetyltransferases (HATs), histone methyltransferases (HMTs), and demethylases, whose coordinated actions establish combinatorial modification patterns across the genome. These enzyme systems do not operate in isolation but instead function within multiprotein complexes that integrate signaling, metabolic, and transcriptional inputs. Dysregulation of this enzymatic balance can therefore result in persistent epigenetic alterations, providing a mechanistic foundation for maladaptive transcriptional programs associated with stress-related psychiatric disorders, including depression (Verma et al., 2022).

### 2.4 Histone modifications and chromatin accessibility

Chromatin remodeling plays a critical role in regulating gene expression, with post-translational modifications (PTMs) on histone tails serving as key modulators of nucleosome dynamics (Chen et al., 2024). While the histone core contributes to nucleosome stability, the flexible N-terminal tails undergo reversible modifications that alter histone–DNA interactions and shift chromatin between active and repressive states (North et al., 2014). Combinatorial PTM patterns—the so-called “histone code”—are interpreted by reader proteins that recruit effector complexes to regulate chromatin accessibility (Kouzarides, 2007; Campos and Reinberg, 2009).

Advances in chromatin profiling technologies, including ChIP-seq and ATAC-seq, now allow high-resolution mapping of histone marks and open-chromatin regions across cell types. ChIP-seq reveals genome-wide distributions of histone modifications and regulatory elements (Nakato and Sakata, 2021), whereas ATAC-seq detects chromatin accessibility changes associated with stress or disease states (Wang et al., 2025). Together, these tools provide essential insight into how histone modifications shape transcriptional regulation in neuropsychiatric conditions such as depression.

### 2.5 Emerging histone acylations: lactylation

Histone lactylation is a recently identified post-translational modification in which lactate-derived acyl groups are covalently attached to lysine residues, forming lysine lactylation (Kla). Glycolysis-derived lactate has been demonstrated to act as a direct precursor for histone Kla, linking metabolic state to chromatin regulation (Pu et al., 2021; Zhang et al., 2023). Increased intracellular lactate, such as during mitochondrial respiratory inhibition, elevates histone lactylation, suggesting that Kla acts as a metabolically sensitive epigenetic signal (Wang et al., 2025).

Histone lactylation promotes transcriptional activation, with enrichment of specific Kla marks (e.g., H4K12la) at promoters of metabolic genes. Physical activity, which elevates systemic lactate levels, may therefore influence neuronal gene expression partially through histone lactylation (Lei et al., 2024). Recent findings indicate that lactate-associated lactylation modulates glial inflammatory states and neuronal synaptic function, supporting its role in neuroplasticity (Yao et al., 2023; Hagihara et al., 2021). In this way, Kla adds an additional layer to histone-based epigenetic regulation, potentially contributing to metabolic–epigenetic coupling relevant to stress responses and neuropsychiatric conditions.

## 3 Histone modifications in the Pathophysiology of depression

### 3.1 Altered histone acetylation in depression

Animal models such as chronic unpredictable stress (CUS) and chronic social defeat stress (CSDS) have been widely used to investigate the epigenetic basis of depression. These paradigms reliably induce depressive-like behaviors—including anhedonia, behavioral despair, and reduced locomotor activity—and are consistently associated with alterations in histone acetylation within brain regions critical for mood regulation, such as the hippocampus and prefrontal cortex (Li et al., 2017; Liu et al., 2014).

Histone acetylation, particularly at lysine residues on histones H3 and H4, generally promotes gene activation by loosening chromatin structure and facilitating the recruitment of transcriptional regulators (Shahbazian and Grunstein, 2007). In depression-related models, reduced acetylation at sites such as H3K9ac and H4K12ac has been associated with decreased expression of key mood-regulating genes, including brain-derived neurotrophic factor (BDNF) and the serotonin transporter (SLC6A4) (Covington et al., 2011; Schroeder et al., 2007). These gene-specific acetylation changes contribute to impaired neuroplasticity and emotional regulation under chronic stress.

In chronic unpredictable stress (CUS) models, acetylation at H3K9ac and H4K12ac sites is markedly reduced in the hippocampus and prefrontal cortex, accompanied by increased expression of histone deacetylase 5 (HDAC5). This hypoacetylation is associated with reduced tyrosine hydroxylase (TH) expression in the locus coeruleus and decreased tryptophan hydroxylase (TPH) activity in the hippocampus—two enzymes essential for dopamine and serotonin synthesis, respectively. These molecular alterations indicate disrupted monoaminergic signaling, a core neurobiological feature of depression (Liu et al., 2014).

Taken together, these findings indicate that chronic stress induces epigenetic suppression of mood-related genes through reduced histone acetylation, in part via HDAC5-mediated mechanisms. Such stress-induced hypoacetylation increases vulnerability to depressive behaviors by impairing neuroplasticity and monoaminergic signaling. These results underscore the importance of histone acetylation dynamics in the pathophysiology of depression and highlight HDAC-related pathways as potential targets for therapeutic intervention.

### 3.2 Histone methylation and repressive marks in depressive phenotypes

In addition to histone acetylation, histone methylation is a key epigenetic mechanism involved in depression. Unlike acetylation, which typically promotes transcription, methylation can either activate or repress gene expression depending on the residue and degree of methylation. Repressive marks such as H3K9me3 and H3K27me3 are particularly relevant, as they are strongly associated with transcriptional silencing and have been implicated in stress-induced depressive phenotypes (Verma et al., 2022).

The histone methyltransferase SETDB1, a major writer of the repressive H3K9me3 mark, has emerged as an important regulator of mood- and cognition-related pathways (Karimi et al., 2011). Although SETDB1 is expressed at relatively low levels in the adult brain, it remains functionally active in both excitatory and inhibitory neurons. Recent studies demonstrate that SETDB1 represses the serotonin receptor gene Htr3a by depositing H3K9me3 at its enhancer region. Loss of SETDB1 results in Htr3a overexpression, increased excitability of cortical GABAergic interneurons, and the emergence of anxiety- and depression-like behaviors in mice. These behavioral abnormalities can be partially reversed by 5-HT3 receptor antagonists, highlighting a functional interaction between histone methylation and serotonergic signaling in mood regulation (Li et al., 2023).

Elevated levels of the repressive H3K27me3 mark, catalyzed by the histone methyltransferase EZH2, have also been observed in chronic stress models. These increases are associated with reduced expression of neuroplasticity-related genes, including CREB and enzymes involved in GABA synthesis (Yizhar et al., 2011). Such epigenetic repression can weaken synaptic transmission and impair cognitive and emotional processing, thereby contributing to heightened vulnerability to stress and depressive phenotypes.

Together, these findings highlight the central role of repressive histone methylation—particularly H3K9me3 and H3K27me3—in shaping stress susceptibility and depressive phenotypes through gene silencing mechanisms. Methyltransferases such as SETDB1 and EZH2 therefore represent promising targets for reversing maladaptive transcriptional repression, providing a mechanistic foundation for epigenetic therapeutic strategies discussed in the following section.

### 3.3 Region-specific epigenetic alterations (PFC, NAc, hippocampus)

A substantial body of evidence demonstrates that epigenetic dysregulation associated with depressive phenotypes exhibits marked regional specificity. The hippocampus and dentate gyrus (DG) consistently emerge as regions of heightened vulnerability. Developmental stress paradigms reveal broad reductions in multiple BDNF transcript variants, increased CpG methylation at BDNF promoters, upregulation of HDAC1/2, and decreased H3K14 acetylation (Zheng et al., 2016), forming a coherent repressive chromatin profile that disrupts hippocampal plasticity and increases susceptibility to mood-related dysfunction.

Environmental exposures during development similarly induce robust and sex-dependent chromatin alterations in the DG. Male animals display increased H3K4me3 and H3K9ac, along with MLL upregulation and KDM5B reduction, whereas females exhibit the opposite pattern, including reduced H3K4me3 and H3K9ac and increased HDAC1/2 expression (Tyler et al., 2015). These findings highlight the sensitivity of the developing hippocampal epigenome to external perturbation and the emergence of stable sex-specific epigenetic trajectories.

In contrast, the prefrontal cortex (PFC) displays a distinct and more selective pattern. Male PFC shows increased H3K4me3 but reduced H3K9ac (Tyler et al., 2015), accompanied by reduced expression of acetyltransferases such as GCN5 and PCAF, indicating impaired transcriptional activation. Females, however, exhibit minimal chromatin alterations.

Collectively, these region-specific chromatin signatures underscore a complex interaction among brain region, developmental stage, and sex in shaping vulnerability to depressive phenotypes.

## 4 Epigenetic therapeutic targets: focus on HDACs and HMTs

### 4.1 HDAC inhibition as a therapeutic strategy

Histone deacetylases (HDACs) are key enzymes that remove acetyl groups from lysine residues on histone proteins, thereby modulating chromatin structure and transcriptional activity. Beyond histones, HDACs also act on numerous non-histone substrates and participate in diverse biological processes, including cell-cycle regulation, DNA-damage repair, metabolic homeostasis, and the development and functional maintenance of the nervous system.

The mammalian HDAC family consists of 18 isoforms categorized into four major classes (Classes I–IV) (Zhang et al., 2024; Glozak et al., 2005; Gallinari et al., 2007; Yang and Grégoire, 2005), as summarized in Table 1 (Vanaja et al., 2018; Johnstone, 2002; Gao et al., 2002; Tong et al., 2002; Hubbert et al., 2002).

**TABLE 1:** Classification and major characteristics of mammalian HDAC isoforms.

Class I (HDAC1, HDAC2, HDAC3, HDAC8)	Mainly localized in the nucleus, responsible for classical transcriptional repression
Class II (HDAC4, HDAC5, HDAC6, HDAC7, HDAC9, HDAC10)	Shuttling between the nucleus and cytoplasm, involved in neural development and synaptic plasticity
Class III (Sirtuins, SIRT1–7)	NAD^+^-dependent deacetylases, closely associated with energy metabolism, aging, and neuroprotection
Class IV (HDAC11)	Shares features of both Class I and Class II HDACs, predominantly localized in the nucleus

In addition to removing acetyl groups, HDACs regulate several other lysine acylations—including depropionylation, demyristoylation, and desuccinylation—which contribute to transcriptional control, metabolic regulation, and cellular stress responses (Wei et al., 2017). Certain isoforms exhibit specialized functions; for instance, HDAC6 primarily targets cytoplasmic substrates such as microtubules, thereby influencing cytoskeletal dynamics and axonal transport (Dompierre et al., 2007). HDAC11 shows strong activity toward long-chain acyl groups and plays a role in lipid metabolism (Moreno-Yruela et al., 2018; Kutil et al., 2018). These noncanonical activities broaden the functional scope of HDACs and underscore their relevance to neurobiological processes implicated in depression.

Under physiological conditions, the balance between HDAC activity and histone acetylation shapes chromatin accessibility and thereby influences gene expression in the nervous system (Zhang et al., 2017). Through this regulatory mechanism, HDACs modulate key neurobiological processes, including neuronal differentiation, synaptic plasticity, learning, and memory formation (Boulasiki et al., 2023; Clapier et al., 2017). These functions highlight the importance of HDAC-dependent chromatin remodeling in brain circuitry relevant to mood regulation.

**FIGURE 2:**
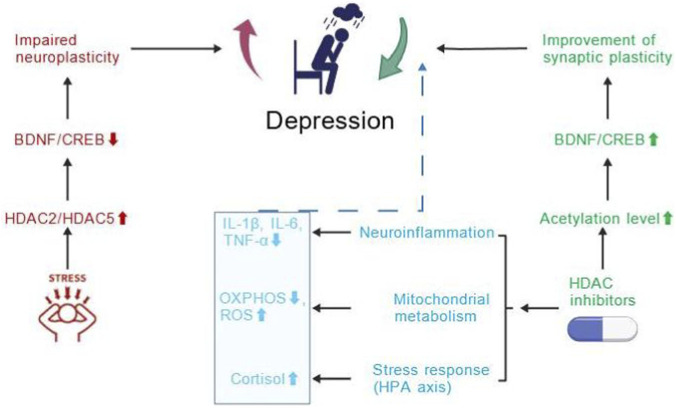
HDAC-mediated mechanisms in depression and therapeutic intervention. Chronic stress increases HDAC2/5 activity, leading to reduced histone acetylation, suppression of BDNF/CREB, impaired synaptic plasticity, and depressive phenotypes. HDAC inhibitors restore acetylation, upregulate BDNF/CREB, and improve plasticity and mood. Beyond transcriptional repression, HDACs also modulate neuroinflammation (↑IL-1β, IL-6, TNF-α), mitochondrial metabolism (↓OXPHOS, ↑ROS), and HPA axis activity (↑cortisol), providing additional pharmacological entry points for antidepressant development (Jiang et al., 2025).

Collectively, these findings indicate that HDAC dysregulation serves as a key molecular link between stress exposure and multiple pathological processes—including impaired neuroplasticity, heightened neuroinflammation, mitochondrial dysfunction, and hyperactivity of the HPA axis—that converge to promote depressive phenotypes. HDAC inhibitors can counteract several of these alterations by restoring histone acetylation and improving neuronal plasticity. Figure 2 provides an overview of these interconnected mechanisms and their therapeutic implications.

### 4.2 HMTs and demethylases in mood regulation

In addition to histone acetylation, histone methylation contributes to chromatin regulation and has become increasingly recognized as a key epigenetic mechanism in depression. Histone methyltransferases (HMTs) catalyze methylation at specific lysine or arginine residues, thereby modifying chromatin conformation and influencing gene expression programs involved in emotional regulation and neural plasticity. Evidence from stress- and depression-related models indicates that dysregulated HMT activity—either excessive methylation or loss of repressive control—can lead to maladaptive behavioral outcomes (Li et al., 2025). These findings highlight HMTs as potential therapeutic targets for modulating transcriptional repression in mood disorders.

Within the Polycomb Repressive Complex 2 (PRC2), EZH2 serves as the catalytic subunit responsible for depositing the repressive H3K27me3 mark and silencing target genes (Blackledge et al., 2020; Laugesen et al., 2019). Recent studies show that PCGF1 enhances EZH2-mediated H3K27me3 enrichment at the MMP10 promoter, reducing the expression of this pro-inflammatory factor. This epigenetic repression attenuates microglia-driven neuroinflammation and alleviates depressive-like behaviors in stress models, underscoring the contribution of EZH2 to inflammation-associated depressive phenotypes and its potential as a therapeutic target (Li et al., 2025).

SETDB1, a histone methyltransferase that deposits the repressive H3K9me3 mark, also contributes to mood regulation through its control of cortical inhibitory interneurons (Karimi et al., 2011) (Schultz et al., 2002; Minkovsky et al., 2014; Matsui et al., 2010). Loss of SETDB1 activity derepresses retroviral-like elements such as RMER21B, enabling aberrant activation of Htr3a enhancers and increasing the number and excitability of Htr3a^+^ interneurons (Li et al., 2023). These alterations disrupt cortical excitatory–inhibitory balance and promote anxiety- and depression-like behaviors. Together, these findings identify the SETDB1–Htr3a axis as a mechanistic pathway linking chronic stress to mood dysregulation and suggest its potential relevance for therapeutic intervention.

Small-molecule inhibitors targeting histone methyltransferases have attracted growing interest in neuropsychiatric research. EZH2-specific inhibitors such as GSK126 and EPZ-6438 (Tazemetostat) can reduce H3K27me3-mediated transcriptional repression and have been shown to alleviate depressive-like and inflammation-associated behavioral abnormalities in preclinical models (Li et al., 2025). These findings provide pharmacological evidence that modulating HMT activity may help reverse stress-induced maladaptive gene regulation and represent a promising therapeutic approach for mood disorders.

Histone demethylases (KDMs) also participate in emotional regulation (Shen et al., 2015). Among them, KDM1A/LSD1 regulates neuronal differentiation and synaptic gene expression via demethylation, and its inhibition enhances neuronal plasticity and improves emotion-related behaviors in preclinical models (Sáez et al., 2015). These findings suggest that balanced histone methylation–demethylation is important for maintaining neural homeostasis in mood disorders.

Epigenetic alterations mediated by H3K27me3 (EZH2) and H3K9me3 (SETDB1) are not unique to depression but also appear in models of schizophrenia and bipolar disorder (Li et al., 2025; Li et al., 2023). This cross-disorder overlap highlights the transdiagnostic potential of targeting histone methylation pathways, suggesting that modulation of HMT or KDM activity may benefit multiple psychiatric conditions. These findings broaden the therapeutic relevance of histone-modifying enzymes beyond depression.

### 4.3 HDAC and HMT modulators beyond gene expression

Traditionally viewed as transcriptional repressors, HDACs are now recognized to regulate broader biological processes beyond chromatin control. Evidence indicates that HDAC activity shapes neuroinflammation, mitochondrial function, and stress adaptation—three interconnected axes that converge on mood regulation. These noncanonical functions interact with acetylation–methylation dynamics and represent additional pharmacological entry points for treating depression.

#### 4.3.1 Epigenetic regulation of neuroinflammation and immune response

Evidence from spinal cord injury models shows that class I HDAC inhibition (e.g., entinostat) suppresses the NLRP3 inflammasome pathway and reduces inflammatory cytokine production, highlighting the HDAC–inflammasome axis as a pharmacologically relevant target (Dai et al., 2021). HDAC inhibitors also demonstrate anti-inflammatory and neuroprotective effects in neurodegenerative models by dampening inflammatory signaling and supporting synaptic and cognitive function (Morrison et al., 2006; Zhou et al., 2018; Kumar et al., 2022). In cellular senescence paradigms, low-dose HDAC inhibitors decrease mitochondrial ROS and cytoplasmic chromatin fragments, thereby reducing SASP-related cytokines and illustrating a mitochondria–inflammation regulatory link (Vizioli et al., 2020).

#### 4.3.2 Inflammatory regulation via NF-κB and NLRP3 pathways

HDAC inhibitors modulate inflammatory pathways beyond transcriptional control (Dai et al., 2025). In UVB-induced photoaging models, SAHA reduces NF-κB–related signaling and pro-inflammatory cytokines, thereby limiting tissue inflammation (Magar et al., 2023; Hu et al., 2022). In spinal cord injury models, entinostat suppresses NLRP3 inflammasome activation and downstream cytokine release (Dai et al., 2021). These findings indicate that HDAC inhibitors exert anti-inflammatory actions through both NF-κB signaling and inflammasome pathways.

#### 4.3.3 Linking anti-inflammatory mechanisms to antidepressant effects

Chronic prenatal stress produces affective abnormalities together with HPA axis hyperactivity and epigenetic disruptions, illustrating the interplay between inflammation, endocrine function, and chromatin regulation in stress vulnerability (Possamai-Della et al., 2023; Glover et al., 2009; Spiers et al., 2014). HDAC inhibitors improve cognitive and behavioral outcomes through anti-inflammatory and plasticity-enhancing actions. In chronic social defeat stress models, entinostat produces antidepressant-like effects (Dai et al., 2021; Covington et al., 2015), reinforcing the link between deacetylation-targeted interventions and stress-related phenotypes.

#### 4.3.4 Mitochondrial function and metabolic remodeling

HDAC inhibitors directly modulate mitochondrial homeostasis. In senescent cells, downregulation of nuclear-encoded OXPHOS genes, reduced mitochondrial membrane potential, and increased ROS activate the ROS–JNK–53BP1–CCF–cGAS–STING cascade, culminating in SASP induction. Low-dose HDAC inhibitors restored OXPHOS gene expression, improved membrane potential, and reduced ROS and CCF/SASP without reversing cell cycle arrest (Vizioli et al., 2020). This suggests that the actions of HDAC inhibitors extend beyond transcriptional regulation to mitochondrial metabolism and suppression of inflammation at its source.

#### 4.3.5 HDAC6, microtubules, and organelle transport

HDAC6, a predominantly cytoplasmic deacetylase, regulates microtubule dynamics and autophagic flux (Kumar et al., 2022). Selective HDAC6 inhibition enhances autophagic throughput, supports axonal growth, and improves organelle transport after injury. HDAC6 also contributes to mitochondrial trafficking and misfolded protein clearance (Perry et al., 2017; Finnin et al., 1999), highlighting the HDAC6–microtubule–mitochondria axis as an important regulatory node beyond chromatin-dependent mechanisms.

#### 4.3.6 Interactions between methylation pathways, metabolism, and stress

Prenatal stress simultaneously elevates DNMT activity and alters H3 acetylation, alongside increased HPA axis reactivity (Possamai-Della et al., 2023), indicating functional interactions between DNA methylation and metabolic/stress pathways (Bagot et al., 2014; Sun et al., 2013). In senescent cells, CCFs are enriched with heterochromatic marks such as H3K9me3 and H3K27me3 (Vizioli et al., 2020), structurally supporting the connection between methylation and inflammation.

#### 4.3.7 Deacetylation and hormonal response in stress adaptation

Animal studies demonstrate that prenatal stress-induced HPA hyperreactivity coexists with alterations in HDAC/DNMT activity, suggesting an interaction between acetylation regulation and hormonal response pathways in stress adaptation (Li et al., 2017; Zhao et al., 2018; Dang et al., 2018). The ability of HDAC inhibitors to improve behavioral outcomes through anti-inflammatory and neuroplastic mechanisms (Dai et al., 2021; Kumar et al., 2022) further supports the concept of an “epigenetic–endocrine–behavioral cascade.”

#### 4.3.8 The framework of “adaptive acetylation response”

Low-dose HDAC inhibitors induce a modest “adaptive acetylation response,” restoring mitochondrial transcription, reducing ROS, and suppressing inflammation without altering cell-cycle arrest (Vizioli et al., 2020). This framework suggests that HDAC inhibitors act upstream by correcting metabolic and inflammatory imbalances. Evidence from NF-κB/mTOR suppression by SAHA and NLRP3 inhibition by entinostat illustrates the cross-tissue and cross-disease relevance of this mechanism (Dai et al., 2021; Dai et al., 2025).

Beyond transcriptional repression, HDACs and HMTs exert multilayered regulatory functions through the modulation of neuroinflammation, mitochondrial metabolism, and stress adaptation. These processes converge to shape neuronal plasticity and vulnerability to stress, thereby providing additional pharmacological entry points for antidepressant development. A concise synthesis of these pathways not only underscores the therapeutic promise of selective HDAC/HMT inhibitors but also highlights the need for improved specificity and context-dependent evaluation in future translational research.

### 4.4 Challenges and limitations of epigenetic therapies

#### 4.4.1 Specificity deficits and off-target effects

HDAC inhibitors often display limited isoform selectivity and off-target activity, which constrains their translational potential in the nervous system (Kumar et al., 2022). SAHA exemplifies broad-spectrum inhibition (Marks et al., 2001; Duvic et al., 2007), whereas entinostat shows greater class I selectivity (Kiany et al., 2020). This contrast highlights a therapeutic dilemma: broad inhibition increases systemic risk, whereas narrow inhibition may reduce efficacy in complex disease networks. Balancing selectivity, safety, and therapeutic impact across disease stages therefore remains a major design challenge.

#### 4.4.2 Safety concerns of broad-spectrum inhibitors

HDAC inhibition may produce neurotoxic or cognitive-impairing effects under certain conditions. Excessive HDAC2 activity is linked to reduced BDNF/c-fos expression and impaired learning and memory (Sun et al., 2019; Lorson et al., 2010), while aberrant HDAC3 expression increases neuronal vulnerability (Kumar et al., 2022). These findings emphasize that dosage, treatment duration, and regional sensitivity critically shape the safety profile of HDAC inhibitors. Consistent with this, Vizioli et al. demonstrated that low-dose HDAC inhibition can improve mitochondrial function and suppress CCF/SASP without reversing growth arrest (Vizioli et al., 2020), illustrating a low-burden strategy targeting upstream pathological pathways.

#### 4.4.3 Reversibility and complexity of epigenetic targets

Epigenetic drugs act on interconnected regulatory networks rather than single linear pathways. TSA and SAHA, for example, do not directly inhibit cGAS–STING signaling or p38/mTOR phosphorylation, but instead restore OXPHOS gene expression and reduce mitochondrial ROS, thereby suppressing CCF and SASP (Vizioli et al., 2020; Khan and La Thangue, 2012; Venkataramani et al., 2010). These findings demonstrate that similar anti-inflammatory outcomes can arise from interventions at different biological levels, indicating that HDAC inhibitor effects extend beyond simple transcriptional repression. Compensatory and counter-regulatory responses further increase mechanistic complexity and challenge therapeutic prediction and biomarker development.

#### 4.4.4 Temporal, regional, and cellular heterogeneity

HDAC expression and functional outcomes vary across tissues, brain regions, and time points. In spinal cord injury models, HDAC1/3 activity differs between the cortex and lesion site (Chen G. et al., 2020; Chen et al., 2018; Chen Y. et al., 2020), and inhibition of specific isoforms such as HDAC8 can improve immune phenotypes without producing behavioral benefits (Dai et al., 2021). These observations illustrate how regional specificity, cell-type differences, disease stage, and outcome measures critically influence the translation of molecular modulation into phenotypic effects. Prenatal stress models further reveal sex-dependent variability in HPA axis reactivity and DNMT/HDAC activity (Possamai-Della et al., 2023), highlighting the complexity of endocrine–epigenetic–behavioral interactions.

#### 4.4.5 Impact of individual differences and pathway interactions on efficacy

Neuroinflammation, metabolic status, and synaptic plasticity jointly influence disease progression. HDAC inhibitors exert neuroprotective effects through both anti-inflammatory mechanisms, such as reduced microglial activation, and non-transcriptional actions, including microtubule acetylation–mediated support of axonal transport and plasticity (Glozak et al., 2005; Chuang et al., 2009; Suh et al., 2010). Studies by Dai and Vizioli show that HDAC inhibition can modulate NF-κB/mTOR signaling and the mitochondria–ROS–JNK–53BP1–CCF axis (Vizioli et al., 2020; Dai et al., 2025), illustrating how upstream metabolic processes and downstream inflammatory responses can be rewired. Baseline factors—including inflammasome and NF-κB activity, mitochondrial function, synaptic plasticity, and HPA axis profiles—therefore shape both the threshold and potential efficacy of HDAC-targeted interventions.

Overall, HDAC-targeted epigenetic therapies are constrained by four major limitations: limited isoform specificity, safety concerns from broad-spectrum inhibition, mechanistic complexity with individual variability, and translational uncertainty driven by temporal, regional, and cellular heterogeneity. Addressing these challenges will require improved isoform selectivity, upstream interventions targeting metabolic–inflammatory interfaces, and greater integration of temporal and individual variability into therapeutic design. These strategies provide actionable directions for advancing HDAC-based treatments for depression and related psychiatric disorders.

### 4.5 Summary

This chapter highlights the roles of HDACs and HMTs in the epigenetic regulation of depression. HDACs influence not only histone acetylation but also non-histone substrates, mitochondrial function, inflammatory signaling, and stress adaptation, underscoring their multilayered regulatory actions. HMTs and demethylases further shape emotional regulation and neural plasticity through locus-specific gene modulation, with EZH2 and SETDB1 central to cross-disorder epigenetic mechanisms. Evidence from animal and cellular models demonstrates that HDAC and HMT inhibitors exert antidepressant and anti-inflammatory effects, supporting their translational relevance.

Remaining challenges include limited isoform selectivity, safety concerns, mechanistic complexity, and individual variability. Future work should prioritize improving isoform specificity, targeting upstream metabolic–inflammatory pathways, and integrating temporal and individual variability into therapeutic design. These strategies will help advance epigenetic interventions for depression and guide the forward-looking perspectives outlined in the next chapter.

## 5 Clinical and translational evidence

### 5.1 Current status of clinical research

Increasing insight into the epigenetic mechanisms of depression has highlighted the translational potential of pharmacological interventions targeting histone acetylation and methylation. Preclinical studies consistently show that HDACs and HMTs regulate stress susceptibility, neural plasticity, cognitive function, and comorbid somatic conditions (Li et al., 2024; Nasehi et al., 2022; Ershadi et al., 2021). For example, HDAC5-mediated H4K12 deacetylation in the hippocampus contributes to stress vulnerability, indicating its value as a potential therapeutic target in susceptible populations (Dai et al., 2023; Li et al., 2024; Ershadi et al., 2021).

The broad-spectrum HDAC inhibitor SAHA exhibits antidepressant effects across multiple animal models, with benefits that extend beyond mood improvement (Nasehi et al., 2022; Ershadi et al., 2021; Chen et al., 2019; Meylan et al., 2016). In cardiovascular comorbidity models, SAHA reduces oxidative stress and metabolic imbalance through downregulation of the NOX4/PGC-1α pathway (Kv et al., 2018; Amini-Khoei et al., 2017). In cognitive impairment models, it increases histone acetylation, enhances BDNF expression, and strengthens antioxidant defenses, thereby improving learning and memory (Chen et al., 2019; Amiri et al., 2017; Sadeghi et al., 2016). In genetically susceptible models of treatment-resistant depression, SAHA inhibits HDAC2, restores histone acetylation and BDNF levels, and reverses antidepressant non-responsiveness (Meylan et al., 2016; Vialou et al., 2013). Together, these findings indicate that HDAC inhibitors may address not only core depressive symptoms but also broader clinical challenges such as cognitive dysfunction, comorbid conditions, and treatment resistance.

Clinically, HDAC inhibitors such as SAHA and entinostat have progressed to phase I/II oncology trials, where preliminary safety and pharmacokinetic profiles have been established (Bare et al., 2024). In depression, however, direct human evidence is still limited, and large-scale randomized controlled trials are lacking. As a result, current clinical relevance is inferred primarily from preclinical studies. Notably, increased HDAC activity and reduced histone acetylation are also reported in bipolar disorder and schizophrenia, suggesting that HDAC/HMT dysregulation represents a cross-disorder mechanism and a theoretical foundation for transdiagnostic epigenetic interventions.

In summary, although HDAC and HMT inhibitors exhibit multidimensional antidepressant potential in preclinical studies and mechanistic evidence supports their translational relevance, clinical development remains in an early phase. Advancing these epigenetic therapies will require rigorously designed trials to evaluate efficacy, safety, and patient-specific applicability.

### 5.2 Peripheral biomarkers

Peripheral blood–derived biomarkers are increasingly valued in epigenetic research on depression because they are non-invasive, repeatable, and suitable for clinical application. Evidence indicates that peripheral blood mononuclear cells (PBMCs) and other circulating molecules can partially reflect central epigenetic states, supporting patient stratification, severity assessment, treatment prediction, and personalized care (Milic et al., 2025; Roy et al., 2017).

#### 5.2.1 DNA methylation and “epigenetic aging”

Clinical studies consistently report methylation abnormalities in stress-related pathways among patients with depression. In PBMCs, NR3C1 hypermethylation with reduced expression and FKBP5 hypomethylation with increased expression correlate with depression severity and suicidal ideation (Roy et al., 2017), supporting HPA-axis dysregulation as a key pathological feature and highlighting PBMC methylation as a potential biomarker (Autry and Monteggia, 2012; Monteiro et al., 2017). Epigenetic age acceleration also shows clinical relevance: measures such as the DunedinPACE clock indicate that depressive symptoms—particularly in women—are associated with faster biological aging, suggesting that peripheral methylation may reflect not only illness severity but also chronicity and recurrence risk (Sorlí et al., 2024; Norbury, 2021; Au and Reece, 2017).

#### 5.2.2 Peripheral molecules and epigenetic relevance

Inflammatory markers such as CRP and IL-6, as well as neurotrophins including BDNF, NGF, and VEGF, are closely associated with depression (Milic et al., 2025). Reduced peripheral BDNF is consistently observed; although levels often increase with antidepressant treatment, their temporal trajectory does not always parallel symptom improvement, suggesting complex regulatory mechanisms (Autry and Monteggia, 2012; Monteiro et al., 2017). Because BDNF expression is linked to histone acetylation, peripheral molecular alterations may partly reflect underlying chromatin states (Li J. et al., 2020; Ribeiro et al., 2021).

#### 5.2.3 Predicting treatment response and enabling stratification

DNA methylation patterns may have value in predicting antidepressant response; for example, altered FKBP5 methylation is associated with both depression severity and SSRI responsiveness (Roy et al., 2017). Patients with elevated inflammatory markers often show better outcomes with anti-inflammatory treatments and poorer responses to psychotherapy, supporting stratified therapeutic approaches (Shi et al., 2022; Jha et al., 2017; Orsolini et al., 2022; Pan et al., 2022). In combination with preclinical evidence that HDAC inhibitors improve cognitive deficits and metabolic imbalance (Nasehi et al., 2022; Ershadi et al., 2021; Chen et al., 2019; Meylan et al., 2016), integrated profiling of peripheral biomarkers and epigenetic signatures may inform personalized pharmacological strategies.

#### 5.2.4 Challenges and outlook

Several uncertainties remain, including the degree to which peripheral epigenetic patterns reflect central pathology, the heterogeneity of existing studies in terms of sample size, analytic platforms, and cell composition, and the lack of multicenter longitudinal validation. Although PBMC DNA methylation and related molecular markers represent promising translational tools, their routine clinical application will require rigorously designed, prospective, and reproducible studies.

## 6 Future Perspectives

### 6.1 Emerging technologies: toward high resolution and manipulability

Future advances in the epigenetic study of depression will increasingly depend on the development and application of high-resolution technologies. Single-cell approaches, including scATAC-seq, scChIP-seq, and single-cell bisulfite sequencing, enable cell type–and state–specific mapping of chromatin accessibility and regulatory element activity. When integrated with transcriptomic and genome-wide association data, these methods facilitate the identification of depression-relevant cell populations and regulatory modules (Kim et al., 2024; Jagadeesh et al., 2022; Kearns et al., 2015; Carleton et al., 2017). Recent studies further indicate that genetic risk for depression is enriched in adult BDNF-positive excitatory neurons as well as in specific developmental cell populations, highlighting both cell-type specificity and developmental-stage vulnerability (Kim et al., 2024).

More broadly, recent large-scale single-cell and single-nucleus epigenomic studies have begun to systematically associate psychiatric risk variants with distinct neuronal and glial subtypes, marking a conceptual shift from bulk tissue–level correlations toward cell-resolved regulatory landscapes relevant to stress-related disorders (Sun et al., 2024).

By anchoring molecular signals to defined anatomical contexts, spatial transcriptomics and epigenomics—such as Slide-seq and MERFISH—provide insight into the spatial organization of inflammatory responses, excitatory–inhibitory balance, and glia–neuron interactions (Wu et al., 2025; Wu et al., 2023). In Alzheimer’s disease research, the integration of spatial and single-nucleus transcriptomics has delineated localized microglial responses surrounding amyloid plaques. A similar framework can be applied to depression-relevant regions, including the prefrontal cortex, hippocampus, and amygdala, to elucidate how stress-related pathways are distributed and coordinated at the circuit level (Chen et al., 2019; Milic et al., 2025). Importantly, integrating spatially resolved molecular data with large-scale neuroimaging frameworks may help bridge cell-specific epigenetic dysregulation and macroscale circuit abnormalities—a gap underscored by recent normative modeling studies revealing substantial interindividual heterogeneity in depression-related brain networks (Sun et al., 2023).

The advent of CRISPR–dCas9–based epigenetic editing has further enabled reversible and locus-specific regulation of genomic regions without altering DNA sequence. By targeting key regulatory elements such as the BDNF promoter, FKBP5, or NR3C1, these approaches allow direct interrogation of causal roles in stress responses and depressive phenotypes (Li K. et al., 2020). At present, such strategies primarily function as proof-of-concept tools for locus-specific mechanistic validation rather than immediate therapeutic applications; nevertheless, they provide a critical experimental bridge from correlative epigenomic associations to causal inference (Sun et al., 2024).

Despite technical challenges—including delivery efficiency, off-target effects, and long-term safety—these emerging technologies collectively form a discovery–localization–validation framework. Together, they establish a methodological foundation for high-resolution stratification and targeted intervention in depression, while also offering broadly applicable tools for psychiatric research across disorders.

### 6.2 Precision medicine: from population to individualized intervention

With the rapid expansion of multi-omics approaches, precision medicine in depression is increasingly shifting from population-level associations toward individualized stratification and treatment matching. In this context, integrative epigenetic profiles—encompassing DNA methylation, histone modifications, and non-coding RNAs—offer a conceptual framework for defining patient subgroups based on underlying regulatory states rather than clinical symptoms alone. Accumulating evidence indicates that methylation patterns of stress-related genes, including NR3C1 and FKBP5, in peripheral blood mononuclear cells are associated with both depression severity and antidepressant responsiveness (Roy et al., 2017). Moreover, large-scale epigenome-wide association studies have identified specific CpG loci, such as RSPO2, IL16, and PRKCI, that correlate with treatment outcomes following SSRI or SNRI therapy, underscoring the potential utility of epigenetic information for patient stratification and therapeutic optimization (Schiele et al., 2024).

With respect to treatment response prediction, epigenetic signatures often exhibit cross-diagnostic convergence rather than disorder-specific exclusivity. Epigenetic alterations in genes such as BDNF, SLC6A4, NR3C1, and FKBP5 have been associated with antidepressant efficacy in major depressive disorder, while comparable patterns have also been reported in bipolar disorder and schizophrenia, supporting their potential utility as transdiagnostic biomarkers (Zhou et al., 2021).

Beyond gene-centered analyses, emerging single-cell and spatial epigenomic approaches further refine precision medicine by linking regulatory variation to specific cell types and neural circuits. For example, single-cell multi-omics profiling of the nucleus accumbens has delineated depression-relevant cell populations and regulatory elements, providing cell-resolved evidence that may inform target prioritization and mechanistic stratification (Bhatia et al., 2023).

Sex differences constitute a critical layer of individual variability in precision medicine for depression. Accumulating evidence suggests that female patients are more likely to exhibit promoter hypermethylation of stress-related genes, including NR3C1 and SLC6A4, which has been associated with increased vulnerability to depression and differential treatment response (Kawatake-Kuno et al., 2021). Consistent with this notion, transcriptomic and methylomic overlap between males and females is limited—often below 10% and, in some cases, directionally opposite—indicating a molecular basis for sexual dimorphism (Mena and Benoit, 2019). In addition, sex-specific interactions between hormonal signaling and histone modifications follow distinct developmental and temporal trajectories, potentially shaping differential windows of vulnerability and clinical phenotypes (Singh et al., 2019). Collectively, these findings underscore that sex should be considered a biological modifier of epigenetic regulation and treatment outcome, rather than merely an epidemiological variable, within precision medicine frameworks.

Collectively, advances in epigenetic profiling are reshaping approaches to precision diagnosis and individualized intervention in depression. Peripheral DNA methylation, cell-resolved epigenomic mapping, transdiagnostic biomarkers, and sex-informed stratification together support a shift from population-averaged treatment paradigms toward biologically informed stratification and personalized therapeutic strategies. Nevertheless, substantial challenges remain in validating the stability, reproducibility, and predictive value of these markers across diverse populations, disease stages, and clinical contexts, underscoring the need for rigorous translational frameworks.

### 6.3 Translational challenges and future directions

Despite the growing promise of epigenetic research in depression, substantial barriers continue to impede the translation of experimental findings into clinical practice. Widely used animal models, which predominantly rely on acute or chronic stress paradigms, have been instrumental in identifying histone modification abnormalities and probing the effects of epigenetic modulators. However, these models incompletely capture the clinical heterogeneity, chronicity, and multidimensional symptom profiles characteristic of human depression (Li et al., 2024; Ershadi et al., 2021). In addition, species-specific differences in epigenetic architecture and regulatory dynamics further constrain the direct extrapolation of preclinical findings to human disease (Wu et al., 2025).

In parallel, many epigenetic modulators, including inhibitors of HDACs and histone methyltransferases, exert broad molecular effects that raise concerns regarding target specificity, off-target activity, and systemic toxicity. In the context of depression—a disorder often requiring long-term treatment—these limitations are particularly salient, as the sustained efficacy and safety of such agents remain incompletely characterized. Consequently, rigorous, large-scale, and multicenter longitudinal studies will be required to evaluate their long-term effects on cognition, metabolism, immune function, and overall clinical outcomes (Schiele et al., 2024).

Clinical trial design poses additional challenges for the translation of epigenetic interventions. Such approaches often require prolonged administration, making long-term safety monitoring, treatment adherence, and attrition management particularly demanding (Fagiolini et al., 2021). Moreover, reliance on symptom-based rating scales alone—such as MADRS or threshold-based metrics derived from the PHQ-9—may be insufficient to capture biologically meaningful treatment effects. Integrating epigenetic and molecular biomarkers as complementary endpoints could provide a more nuanced assessment of therapeutic response.

Patient heterogeneity represents a further major obstacle to clinical translation. Variability across diagnostic subtypes, sex, genetic background, and baseline biological states is likely to influence both treatment response and tolerability. Accordingly, trial designs incorporating stratification strategies based on these factors may be critical for improving signal detection and determining the success of future epigenetic therapies (Kawatake-Kuno et al., 2021; Mena and Benoit, 2019).

Moving forward, addressing these translational challenges will require an integrated and multidisciplinary framework that bridges neuroscience, immunology, and metabolism. Neuroscience provides insight into synaptic plasticity and circuit remodeling; immunology elucidates interactions among inflammation, epigenetic regulation, and depressive pathology; and metabolic research highlights the influence of energy homeostasis and mitochondrial function on chromatin dynamics. Within this framework, next-generation antidepressant strategies are likely to move beyond single-target paradigms toward rationally designed, multi-mechanism approaches that combine epigenetic modulators with established pharmacological, psychotherapeutic, and lifestyle-based interventions.

Equally important, effective translation will depend on precision stratification strategies that integrate genetic background, sex-specific molecular trajectories, environmental exposures, and baseline biological states. Given the transdiagnostic features of epigenetic dysregulation, such approaches may be applicable not only to major depressive disorder but also to related psychiatric conditions, including bipolar disorder and schizophrenia.

Ultimately, the establishment of a neuro–immune–metabolic framework for epigenetic intervention may provide a coherent pathway for translating mechanistic insights into clinically actionable strategies, thereby informing the development of next-generation treatments for depression and related disorders (Wu et al., 2025).

## 7 Conclusion

Major depressive disorder is a complex and heterogeneous condition shaped by the dynamic interplay among genetic susceptibility, environmental stressors, and epigenetic regulation. Accumulating evidence indicates that histone acetylation and methylation play central roles in governing neural plasticity, stress responsiveness, and emotional regulation. In this context, histone deacetylases and histone methyltransferases emerge as key molecular regulators that link environmental exposures to persistent transcriptional alterations relevant to depressive pathology.

Importantly, the biological functions of these enzymes extend beyond canonical chromatin remodeling. HDACs participate in the regulation of neuroinflammatory signaling, mitochondrial metabolism, and stress adaptation, while HMTs and histone demethylases mediate locus-specific transcriptional control across neural circuits. Preclinical studies across cellular and animal models consistently demonstrate that modulation of these epigenetic pathways can alleviate depression-like phenotypes and promote neuroplasticity, highlighting their relevance not only to depression but also to broader, transdiagnostic psychiatric processes.

Despite these advances, substantial challenges remain. Limited isoform specificity, off-target effects, mechanistic complexity, and pronounced interindividual and stage-dependent variability constrain the clinical translation of epigenetic therapies. Robust clinical evidence is still lacking, underscoring the need for well-designed, long-term, randomized trials. In parallel, peripheral epigenetic markers—particularly DNA methylation signatures—offer promising avenues for patient stratification and treatment prediction, although their reproducibility and clinical robustness require further validation.

Looking ahead, emerging technologies including single-cell and spatial epigenomics, together with CRISPR-based epigenetic editing, are poised to refine the resolution at which depression-related regulatory alterations can be interrogated. Integrated within multi-omics and precision medicine frameworks, these approaches may enable the construction of individualized epigenetic profiles that inform stratified and context-aware interventions. Moreover, the convergence of neuroscience, immunology, and metabolism provides a critical foundation for the rational development of next-generation antidepressant strategies.

In conclusion, epigenetic research has substantially deepened our understanding of depression pathophysiology while opening new translational possibilities. Continued integration of mechanistic insight, technological innovation, and clinical rigor will be essential for translating epigenetic discoveries into safe, effective, and personalized treatments for depression.
